# An Estimation Model of Urban Land Accessibility

**DOI:** 10.3390/ijerph18031258

**Published:** 2021-01-30

**Authors:** Wei Wang, Jian Chen, Zhiyuan Wang, Jun Chen, Wen Cheng, Zihao Zhou

**Affiliations:** 1 School of Transportation, Southeast University, Nanjing 211189, China; wwang@seu.edu.cn (W.W.); 220183102@seu.edu.cn (J.C.); chenjun@seu.edu.cn (J.C.); 220183163@seu.edu.cn (Z.Z.); 2 School of Architecture, Key Laboratory of Cold Region Urban and Rural Human Settlement Environment Science and Technology, Harbin Institute of Technology, Harbin 150001, China; hit_chengwen@hit.edu.cn

**Keywords:** grid measurement, comprehensive accessibility, urban planning, geographic information system

## Abstract

Given that there are no practical quantitative indicators of traffic conditions for facility location selection in the process of urbanization, this article proposes a comprehensive accessibility index of location and its measurement method. Urban land is rasterized using GIS to obtain the grids, and the road network data are used to calculate the external accessibility and internal accessibility of the grids. The external accessibility and the internal accessibility of a grid are combined to obtain the comprehensive accessibility of the location. The comprehensive accessibilities of grids are measured for Zhicheng, an urban area in China. The results show that the pattern of gradual spatial changes in the comprehensive accessibility of the grids in Zhicheng is highly consistent with the urban land’s spatial development trend, which verifies the feasibility and accuracy of the comprehensive accessibility measurement method. On one hand, the comprehensive accessibility of the grid is more portable than the accessibility of a single point and can be calculated in batches. On the other hand, it is more specific than the regional accessibility and better guides the location selection of urban facilities.

## 1. Introduction

In recent decades, cities in China have expanded significantly. The continuous establishment of high-tech zones and new districts has enabled urban construction to quickly break through the boundaries of the old city area. According to a set of data from the National Bureau of Statistics of China, the domestic urban built-up area was 54 thousand square kilometers by the end of 2016, which indicated an increase of 47 thousand square kilometers from the end of 1981 [1]. Facing increasing demand, urban planning of expanding cities becomes a difficult problem to solve. Normally, large-sized urban facilities, such as integrated transportation hubs, will be systematically planned by the city authorities and planners during urban expansion. However, small and medium-sized facilities used to help urban residents live and work must also be carefully laid out and constructed in the same period. The location selection of these facilities is mainly based on indicators such as topography and land use type, but the traffic conditions in the location is not quantified. As a result, the location of the facilities was not convenient for the people. Consequently, new road infrastructure had to be built so that the facilities could provide better services, resulting in a waste of resources. The fundamental problem is that it is hard to determine the advantages and disadvantages of the traffic conditions of any location chosen for the facilities due to the absence of a method for measuring the traffic conditions of the location. Therefore, this article evaluates the measurement methods of traffic conditions in urban land and establishes the foundation for the proper planning and location selection for important facilities of public services, such as medical facilities and educational facilities.

Accessibility is an indicator for measuring the traffic conditions of a single facility or a certain type of facility. It measures the extent to which a facility is accessible. To measure the traffic conditions of a certain region, the indicator of road network density, which refers to the ratio of the total length of the road network in a certain region to the area of this region, is commonly used [2,3].

Based on the traditional concept of accessibility introduced above, the concept of comprehensive accessibility of a grid is proposed to reflect the traffic conditions of the grid. Unlike the traditional accessibility, the comprehensive accessibility of a grid does not take a single point as the research object. It uses the geographical information system (GIS) for rasterization. Taking a grid cell, i.e., a grid, after the rasterization as the research object, it comprehensively considers the external accessibility of the grid (the accessibility of other grid cells to this cell) and the internal accessibility (the degree of accessibility within the cell) to measures the degree of accessibility of a grid. In this article, we aim to propose a rational model for the estimation of comprehensive accessibility. This model is able to give an integrated and visual result of the comprehensive accessibility of different grids within an urban district.

## 2. Literature Review

Accessibility is the most commonly used traffic condition, and many estimation methods have been derived since the concept was proposed. The concept of accessibility was first proposed by Hansen [4]; accessibility was defined as the opportunity for nodes in the traffic network to interact with each other. A gravity model was also used to study the relationship between accessibility and urban land use [4]. In the field of transport planning, accessibility is widely regarded as the ability of people to reach a place, or the degree to which a place is accessible to people when a means of transportation with a given capacity is the main mode of travel. Geurs and Van Eck [5] defined accessibility “as the extent to which land-use and transport systems enable (groups of) individuals to reach activities or destinations by means of a (combination of) transport mode(s).” It can be seen that although there is a slight difference in the conceptual definitions of accessibility, the essential characteristics of accessibility are the same, which can be summarized as follows: (1) The calculated accessibility value itself is not of substantial significance. Only when in a specific area, the accessibility values of various locations are compared, will it have the ability to explain. Accessibility is not the nature of a place itself but reflects the position or location of the place in the entire area. (2) If the accessibility between two points is not one-way, the accessibility value has two-way equivalence, that is, the accessibility value of A to B is equal to that of B to A [6].

The estimation methods of accessibility can be summarized into four categories. They are summarized in Table 1.

(1) Based on spatial barrier

The physical isolation of two places is used as a measure of the accessibility of one place from the other: for the same location, a location farther away from it has less accessibility than a location closer to it. If the connection between two locations is not unidirectional, then accessibility is reflexive: that is, accessibility from A to B is equal to accessibility from B to A [10]. Physical distance, time, or cost can be used to measure the degree of separation between two locations. On a broader scale, the overall accessibility of a place can measure the accessibility of a place to all other places in some enclosed space [9].

(2) Based on topological structure

Usually the topology of accessibility is achieved by considering only the presence and number of links rather than the absolute distance between network vertices, but this is not strictly necessary. Taaffe and Gauthier [8] briefly and in detail describe the approach to accessibility by using either the relevant number of vertices or the more comprehensive Shimbel exponent, respectively. The advantage of this method is that it can do a lot of computation.

(3) Based on gravity model

The measurement method based on gravity model is the most mainstream accessibility measurement method. Hansen [4] proposed to realize it by combining the actual internode distance on the network with the measurement of attraction of other nodes. The attractiveness of a particular node (destination) decays in relation to its distance from the reference point (origin), as a measure of the relative accessibility of that destination under the current attractiveness. Although accessibility measurements based on gravity models have been very popular, their application has not been very smooth. The reasons are as follows: First, each study redefines the opportunity as the discount rate for the distance that must be reached [11]. Second, the difficulty in measuring accessibility by gravity method is that gravity measurement is sensitive to area size and zonal configuration, as well as the selection of attraction variables and the parameter value of travel impedance term.

(4) Based on accumulative opportunities

This kind of accessibility is calculated based on the number of locations that can be reached from a given origin within a given travel distance or time. Sherman et al. [9] studied the inverted measurement of cumulative opportunity and calculated the number of regions within a given travel distance and time from a location. An obvious flaw in the cumulative chance measure is the arbitrariness of the iso-temporal (or iso-distance) selection, and the fact that there is no difference between the chance of being adjacent to the origin and the chance of being just within the iso-temporal range.

Accessibility is mainly used to determine the traffic convenience of a certain type of facility or a specific facility or a certain area. Liu et al. [12] analyzed the accessibility of hospitals in Chengdu based on real-time traffic conditions. By constructing a comprehensive evaluation model for accessibility of urban tourist attractions, Xie et al. [13] comprehensively evaluated the accessibility of A-level tourist attractions in Wuhan. Liu et al. [14] measured the comprehensive traffic accessibility of counties in the Beijing–Tianjin–Hebei region. Yu et al. [15] established a comprehensive traffic accessibility evaluation system for Shandong Province and analyzed the relationship between regional comprehensive traffic accessibility and economic development. The current point-oriented accessibility estimation is very inflexible, and a location must be specified first before its accessibility is calculated, so its portability is poor; further, it cannot be calculated in batches. The area-oriented accessibility covers too large a scope, which makes it impossible to guide the location selection of urban facilities in urban land. In addition, the estimation of accessibility has failed to consider the road network density, which is an important traffic indicator.

## 3. Methods

In the Introduction section, we have mentioned that the comprehensive accessibility considers both external and internal accessibility of a grid. When calculating the external accessibility of a grid, the center of the grid is regarded as the traffic starting destination or origin to measure the accessibility between grids. The internal accessibility is measured by the road network density. Therefore, the comprehensive accessibility of a grid has the following advantages: (1) it has better portability than location accessibility, (2) it is more specific than regional accessibility, providing better guidance for the location selection of facilities, (3) batch calculation is possible, and (4) internal accessibility and external accessibility are both considered.

**Figure 1:**
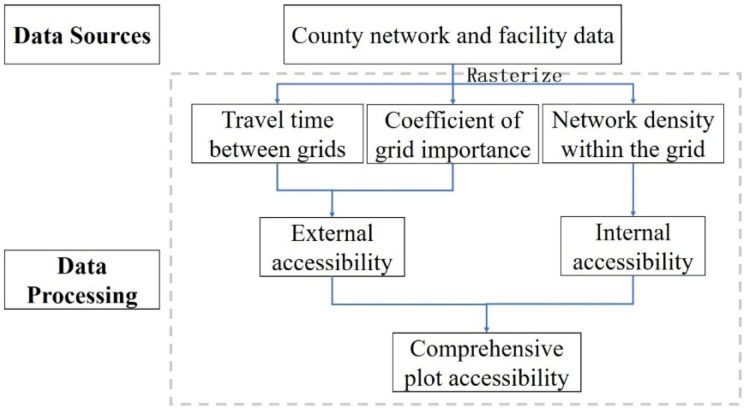
Flow chart of grid accessibility estimation.

First, after the appropriate length is selected, GIS (ESRI, Redlands, CA, USA). is used to rasterize the urban land. According to the travel time between the grid cells and the importance coefficient of each cell, the values of the accessibility of grid i to all other grids are calculated. The values of the external accessibility of grid i to all other grids are added to obtain the external accessibility of grid i, which is divided by the maximum external accessibility value of all grids to obtain the relative external accessibility of grid i in the range of 0–1. Internal accessibility is usually measured by road network density, which also needs to be processed into relative values. Finally, the internal and external accessibilities were considered together to obtain the comprehensive accessibility of the grid. The estimation flow chart is shown in Figure 1.

### 3.1. Estimation of the Relative External Accessibility of a Grid

(1) Select the appropriate rasterization side length and use GIS to rasterize urban land and divide them into grids of the same size. The size can be determined according to different research objects or specific purposes.

(2) Determine the importance levels $w_{1i}$ and $w_{2i}$ (collectively referred to as $w_{i}$) of grid i (i.e., grid cell *i*) as the destination and origin, respectively. The importance levels are mainly related to the number of various facilities in the grid. The estimation process is as follows:

(1) $$R_{i}=\sum \lambda_{j}N_{j}$$

(2) $$w_{i}=\left\{\begin{array}{ll} \frac{R_{i}-1}{R_{\max}-1}+1 & \partial j,N_{j}\neq 0\\ \;1 & \forall j,N_{j}=0\; \end{array}\right.$$

Here, $\lambda_{j}$ is the importance of type *j* facility that has an impact on traffic generation and attraction, the lower limit for $\lambda_{j}$ is 1. $N_{j}$ is the number of type *j* facility that needs to be considered when grid i is the traffic origin and destination, and $R_{i}$ is the intermediate variable of importance for grid i. For the subsequent calculation of external accessibility *A*, the value of $w_{i}$ is fixed in the range of 1.0–2.0 through standardization. If there is no important public facility in grid i ($\forall j,\;N_{j}=0$), then $w_{i}=1$; otherwise the value of $w_{i}$ will be in the range of (1, 2) based on the value of $R_{i}$*,* which is greater than 1.0 ($\exists j,\;N_{j}\neq 0$).

(3) Calculate the shortest time $T_{ij}$ required to travel from the center point of grid i to the center point of grid j. GIS can be used to calculate the shortest path and the shortest time based on the road network data.

(4) Calculate the external accessibility $A_{ij}$ between grid i and grid j by multiplying $w_{1i}$ with $w_{2j}$ and the distance decay function $f\left(T_{ij}\right)$.

(3) $$A_{ij}=w_{1i}w_{2j}f\left(T_{ij}\right)$$

(5) Calculate the external accessibility of grid i by taking the sum of the values of accessibility between grid i and all other grids.

(4) $$A_{i\;\;external}=\sum_{j=1,j\neq i}^{n}A_{ij}$$

(6) Calculate the relative external accessibility of grid i:

(5) $$A_{i1}=\frac{A_{i}}{A_{\max}}$$

### 3.2. Estimation of Relative Internal Accessibility of a Grid

Internal accessibility mainly considers the internal road network density, which is measured by the road network density:

(6) $$A_{i\;\;external}=\frac{L_{i}}{S_{i}}$$

(7) $$A_{i2}=\frac{A_{i}}{A_{\max}}$$

Here, $L_{i}$ is the total length of the road network inside grid i, $S_{i}$ is the area of grid i, and $A_{i2}\;$ is the relative accessibility inside grid i.

### 3.3. Estimation of Comprehensive Accessibility

Here, $\gamma_{1}\;$ and $\gamma_{2}$ are coefficients and $\gamma_{1}$ + $\gamma_{2}\;$ = 1; $\gamma_{1}$ and $\gamma_{2}$ are related to the planning region and the planning purpose. By multiplying internal accessibility and external accessibility by different coefficients to reflect the varying degrees of importance of internal accessibility and external accessibility in the urban planning of the region, the absolute value of comprehensive accessibility is obtained, and $A_{i}$ is standardized to obtain the comprehensive accessibility:

(8) $$A_{i}=f\left(A_{i1},A_{i2},\gamma_{1},\gamma_{2}\right)$$

(9) $$A_{i\;\;comprehensive}=\frac{A_{i}}{A_{i,\max}}$$

## 4. Case Study

Zhicheng, located in Zhejiang Province, China, is an urban area which is undergoing fast expansion. It is a typical example of China’s urbanization process. Focusing on structural adjustment, Zhicheng has vigorously implemented the strategies of “industrial strength city,” “open development city,” and “ecological excellence city,” which has further promoted the sustainable, rapid, and healthy development of economy and society. Zhicheng has a population of 49,400 and covers an area of 88 square kilometers. It has jurisdiction over 6 directly affiliated community committees and 15 administrative villages.

This article used Zhicheng to analyze the comprehensive accessibility of its grids.

### 4.1. Description of Zhicheng’s Urban Road Network Data

The data used in this study came from the National Road Network Data in shapefile (.shp) format, including traffic data of various levels of road networks and stations, as well as the distribution data of various service facilities, such as government, transportation node, medical facility, and educational facility. These facilities will influence the importance of the grids in which they are located. That is to say, the traffic volume to or from these grids is more than that of the grids without those service facilities. The data are imported into GIS. The specific data are shown in Table 2.

### 4.2. Preprocessing and Rasterization of Zhicheng Data

**Figure 2:**
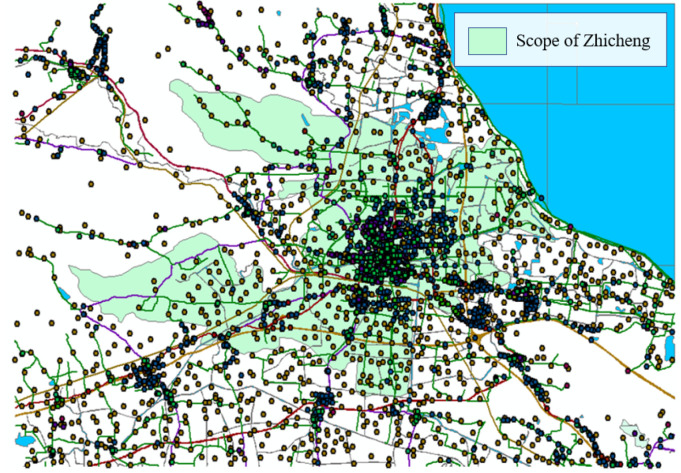
Preview of the map of Zhicheng.

A search was done for Zhicheng on OpenStreetMap, and the urban land boundary was exported. After being converted to .shp format, it was imported into GIS. If only the borders of Zhicheng were cut out for analysis, it might cause inaccurate accessibility of some grids on the borders of the urban land due to the lack of road networks outside the borders. Therefore, after a proper expansion of the range, the data within the expanded range were exported from the national data. The map of Zhicheng in ArcMap is shown in Figure 2, where the green area represents the scope of Zhicheng.

**Figure 3:**
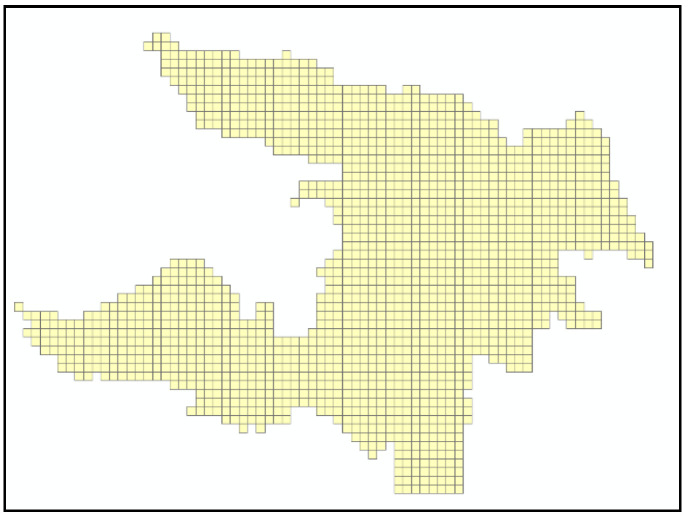
Zhicheng’s grids after rasterization.

**Figure 4:**
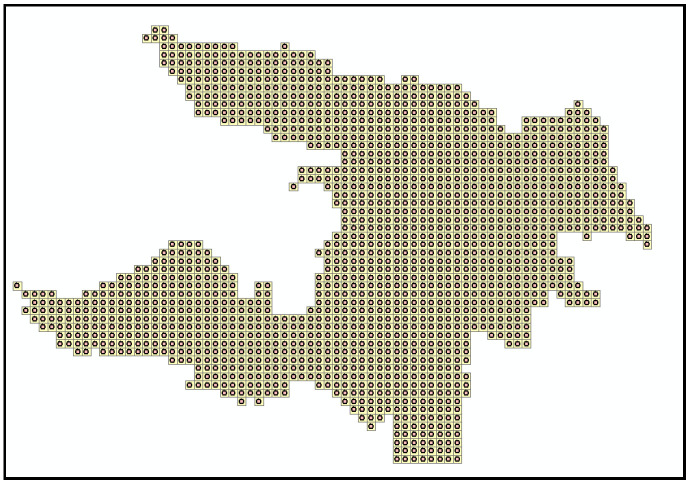
Zhicheng’s grids and their center points after rasterization.

The map was projected to the WGS 1984 World Mercator coordinate system. For an urban area like Zhicheng, it is recommended to divide the urban land into grids of 10~35 hectares. In this case, we decide to make every grid as a square with 400-m-long sides. That is to say, the entire area was rasterized with a cell of 400 m × 400 m, resulting in a total of 1669 cells, or 1669 grids (Figure 3). After rasterization, the center point of each cell was generated to calculate the external accessibility of each cell (Figure 4).

### 4.3. Estimation of the Relative External Accessibility of a Grid

**Figure 5:**
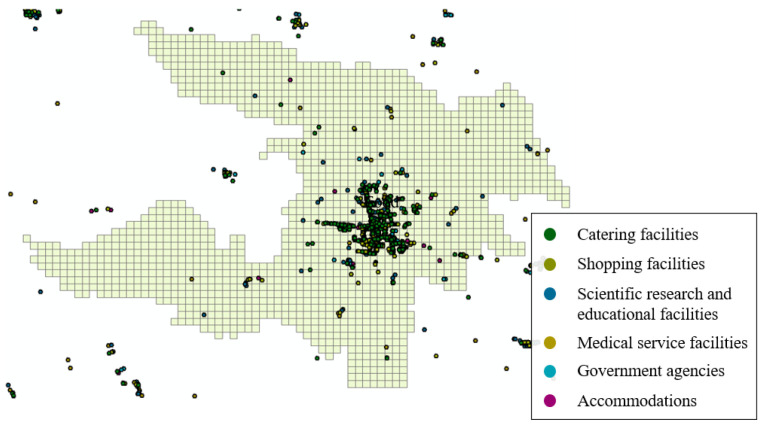
Distribution of related facilities.

The importance coefficient $w_{i}$ was determined by the number of various facilities on grid i. It is an indicator that reflects the importance of grid i in calculating accessibility. We can observe from Formula (2) that the range of $w_{i}\;$ is 1.0–2.0. The facility types mainly included scientific research and education facilities, shopping facilities, medical service facilities, government agencies, catering facilities, and accommodations. The distribution of related facilities is shown in Figure 1 and Figure 5.

In the estimation of accessibility, $w_{1i}$ is the importance of grid I as a traffic destination, and $w_{2i}$ is the importance of grid i as a traffic origin. Assuming that all types of land use have the same impact on the traffic origin and destination, that is, $w_{1i}=w_{2i}$ (collectively referred to as $w_{i}$). $w_{i\;}$ is calculated according to Formulas (1) and (2).

**Figure 6:**
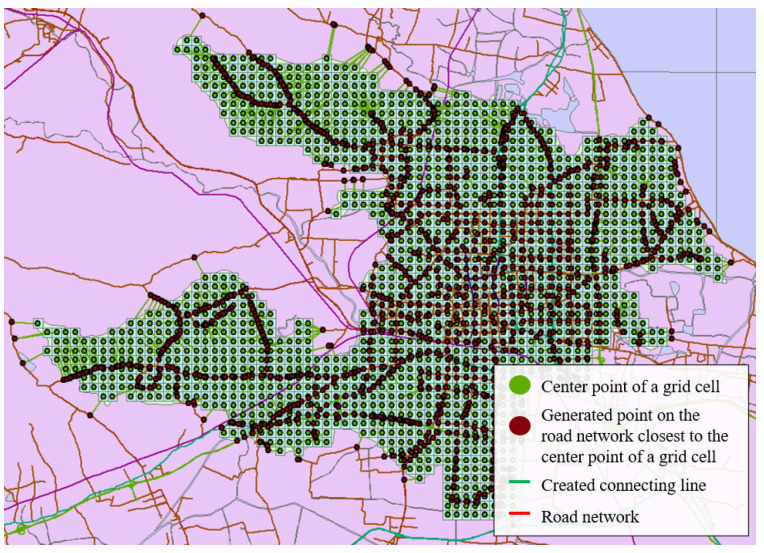
Zhicheng’s road network model.

**Figure 7:**
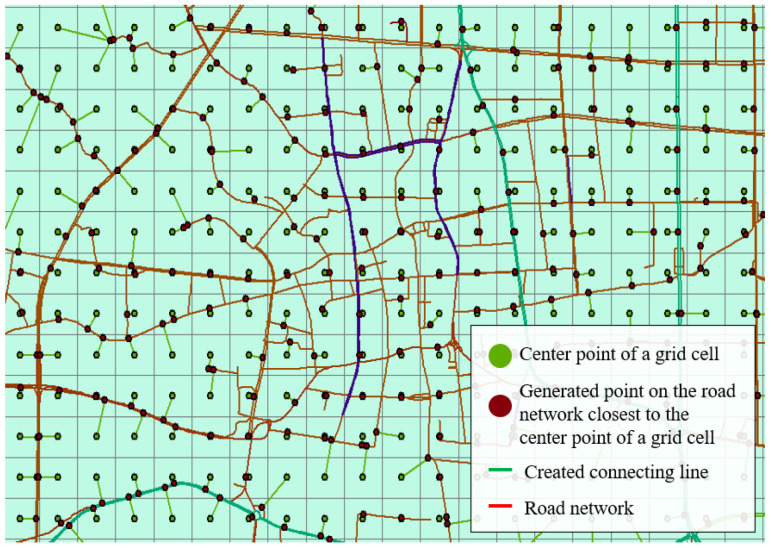
Details of Zhicheng’s road network model.

The shortest time required to travel from the center point of grid I to the center point of grid j, $T_{ij}$, was then calculated. It was assumed that the travel started from the center point of each cell, and the road closest to the center point was chosen. Thus, the point on the road network closest to the center point was found to draw the nearest point on the road network corresponding to each center point on the map, and the nearest points on the road network were connect to the center points to generate branch roads, which connected the central point of each grid cell to the road network, as shown in Figure 6 and Figure 7.

The travel time between grid cells was calculated to create the “origin to destination” (OD) cost matrix. The optimal path was calculated using GIS, and the shortest travel time was also calculated:

(10) $$t_{OD}=\sum_{i}\sum_{j=1}^{n_{i}}\frac{L_{i}}{v_{i}}$$

Here, $t_{OD}$ is the shortest travel time between the origin and destinations i refers to roads of various levels, including expressway, highway, arterial road, urban road, rural road, branch road; $L_{i}$ is the length of a road of level i in the shortest-time route; $n_{i}$ is the number of roads of level i in the shortest-time route; $v_{i}$ is the speed limit of roads of level i in the shortest-time route. The values of speed limit are taken from the local road design standard and are listed in Table 3. Among them, the speed limit for branch road is allowed within the range of 15–20 km/h, so we take 18 km/h as the average standard.

The speed limit field was filled for the routes in GIS, and the time required for the paths between various grid cells was found. The time required for the paths between some grid cells is shown in Table 4.

The total travel cost and the interaction between the origin and destination are directly related to the distance between the origin and destination. The interaction decays with the increase in distance, time consumption, or cost. Therefore, the distance decay function was used to characterize the relationship between accessibility and travel time. The distance decay function uses a common negative power function in the analysis [16]:

(11) $$f\left(d_{ij}\right)=d_{ij}{}^{-\alpha }$$

(12) $$A_{ij}=w_{1i}w_{2j}f\left(d_{ij}\right)$$

(13) $$A_{i\;\;external}=\sum_{j=1,j\neq i}^{n}A_{ij}$$

(14) $$A_{i1}=\frac{A_{i}}{A_{\max}}$$

Here, $d_{ij}$ is the generalized distance between the origin and destination; α is the influence of distance, and α is 1 when the value of $d_{ij}$ is $T_{ij}$; $A_{ij}$ is the external accessibility from i to j; *A_i external_* is the external accessibility of a starting point, which is the sum of the external accessibility values of all pairs of origins and destinations containing that starting point; $A_{max}$ is the maximum value of the external accessibility values of all grids; and $A_{i1}$ is relative external accessibility.

**Figure 8:**
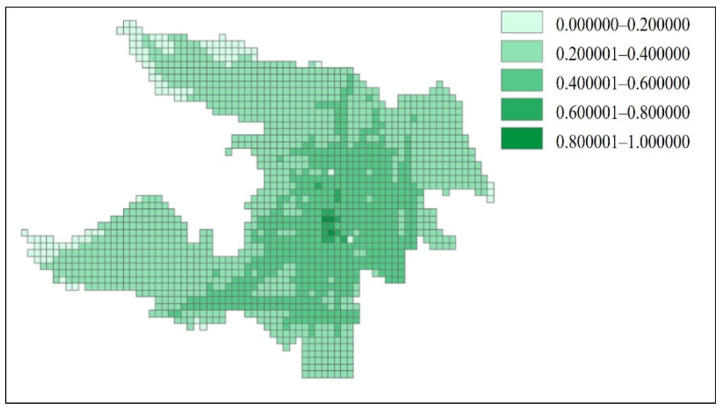
Visualization of the relative external accessibility of Zhicheng.

In GIS, different color blocks were used to indicate different external accessibility ranges in Figure 8.

The external accessibility ranges shown in Figure 8 are able to correspond to the direction of the road network (Figure 6). This shows that the external accessibility estimation is accurate.

### 4.4. Estimation of the Relative Internal Accessibility of the Grid

**Figure 9:**
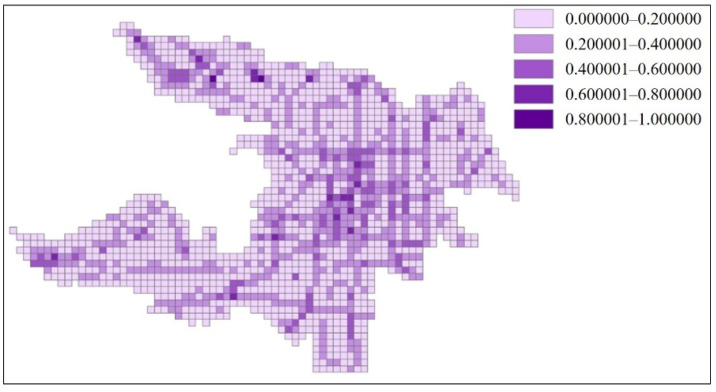
Visualization of the relative internal accessibility of Zhicheng.

Internal accessibility was measured by the internal road network density, and it was calculated according to Equations (6) and (7). The different accessibility ranges were drawn with different color blocks in the GIS. The visualization of internal accessibility is shown in Figure 9.

The internal accessibility is only related to the density of the internal road network of the grid, so the internal accessibility indicated in Figure 9 closely fits the distribution of the road network (Figure 6).

### 4.5. Estimation of Relative Comprehensive Accessibility of the Grid

Comprehensive accessibility is a combination of external accessibility and internal accessibility; external accessibility is jointly restricted by the rasterization scale, the importance coefficient of traffic origin and destination, and the spatial barrier function. Additionally, the key to the final comprehensive accessibility is how to determine the impact of internal accessibility and external accessibility on comprehensive accessibility under different circumstances.

The correlation between the internal accessibility and external accessibility was analyzed on the scale of the grid side length of 400 m in SPSS (IBM, Armonk, NY, USA) in Table 5. The Pearson correlation from the analysis is 0.408, while the two-sided significance is 0.000 (<0.01), indicating that the internal accessibility and the external accessibility are positively correlated at the 0.01 level. This is consistent with objective knowledge.

**Figure 10:**
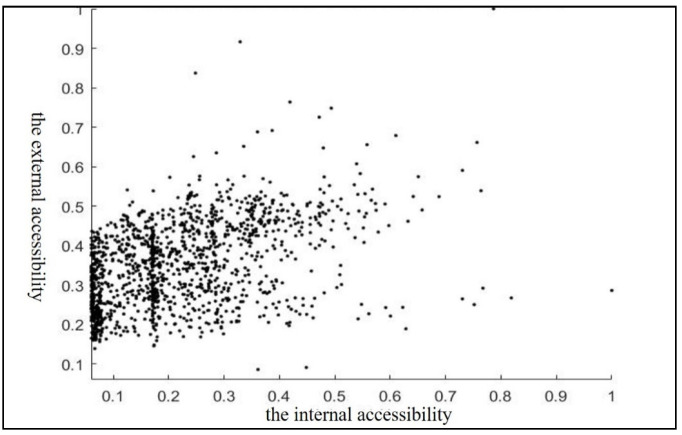
Diagram of correlation between the external accessibility and the internal accessibility of the grids of Zhicheng.

Analyzing the scatter grid (Figure 10) shows that, in the interval of 0.1–0.5, the data are relatively scattered, showing no correlation between the external accessibility and internal accessibility, so it is necessary to study the external accessibility and the internal accessibility at the same time. Since the grid side length is 400 m, anywhere inside the grid can be accessed by walking or riding a bicycle, and the importance of the internal accessibility is weaker than that of the external accessibility. Therefore, $\gamma_{1}$ was set to 0.8 and $\gamma_{2}$ to 0.2, and the two values were substituted into Equation (8) to obtain the absolute comprehensive accessibility $A_{i}$. Finally, $A_{I}$ was standardized using Equation (9) to obtain the relative comprehensive accessibility. The visualization of the comprehensive accessibility in GIS is shown in Figure 9.

**Figure 11:**
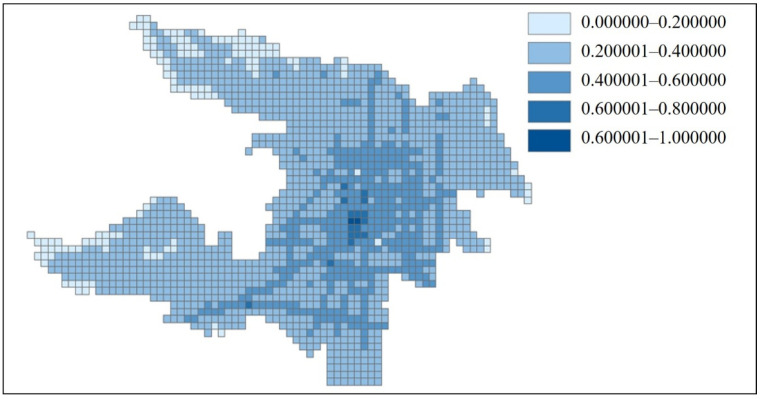
Visualization of the comprehensive accessibility of Zhicheng.

The final comprehensive accessibility (Figure 11) is consistent with the spatial development trend of Zhicheng. Grids with a comprehensive accessibility of 0.8–1.0 are located in the core of the central urban land, a location that has very convenient transportation and maximum number of facilities. Grids with a comprehensive accessibility of 0.6–0.8 are located in the inner circle of the central urban land. Grids with a comprehensive accessibility of 0.4–0.6 are located in the outer radial zone of the central urban land, with convenient transportation and a large number of facilities, or located along the direction of important transportation facilities, with convenient transportation and a small number of facilities. Grids with a comprehensive accessibility of 0–0.2 are located at the periphery of the urban land.

## 5. Discussion

The existing method for accessibility estimation can be divided into two types. The first type is point-oriented. In this type of estimation, researchers must firstly specify a certain location or facility before calculating, and the method is not flexible enough. The other type is region-oriented, but this area of region is usually very large, which leads to its estimation granularity is not fine enough. The comprehensive accessibility estimation method proposed in this article can divide urban land into small-scale and large-quantity grids and analyze all grids in the region uniformly. The estimation of comprehensive accessibility integrates the accessibility inside and outside the grid, while the previous researches mainly calculate the external accessibility, thus we name our proposed accessibility as comprehensive accessibility. As a result, our comprehensive accessibility can more accurately and comprehensively reflect the accessibility of grids. Focusing on travel time, the external accessibility emphasizes the convenience of transportation between grids. The calculation method for external accessibility is optimized on the basis of the most widely applicable gravity-based model, and the quantity and importance of major service facilities inside a grid are used to measure the attractiveness of the grid. It breaks through the limitations of the attractiveness measurement in the previous models that are subjectively calculated and difficult to determine. At the same time, in the process of calculating external accessibility, we take into account the differences in the vehicle speeds of different road levels, and calculate the travel time instead of the travel distance, which is in line with human’s psychological sensitivity to travel time. Internal accessibility is another component of comprehensive accessibility, it emphasizes the accessibility within every grid, which is measured by road network density.

This comprehensive accessibility estimation method has the following advantages: (1) compared with point accessibility, it has better portability; (2) it is more specific than regional accessibility, and provides better guidance for the location selection of facilities; (3) it can be calculated in batches, with all grids calculated, can be used for comparison, and is comprehensive; (4) it takes into consideration the internal accessibility and external accessibility; (5) the results can be intuitively and clearly visualized.

Although this article mainly uses Zhicheng as a case, other researchers may estimate the comprehensive accessibility of each grid of any other urban land by importing the basic data there into GIS and then following the method proposed in this article. Of course, some parameters such as grid size, travel speed, importance weights of various existing facilities, and so on need to be set by researchers according to their specific research objects.

The method proposed in this paper also has some limitations. Firstly, this method is suitable for reconstruction or expansion of urban land, but it cannot guide the location selection of facilities when building a brand-new city. Since the proposed method needs to refer to the existing infrastructures and facilities when estimation the comprehensive accessibility, the model cannot get enough input data for a brand-new urban land without any existing infrastructure or facility. Secondly, this method mainly refers to static data such as road network, and does not take into account the change of different travel time between originations and destinations with different transports or in different time periods. In future study, we may try to obtain various aspects of data to improve the calculation accuracy and make the estimation of accessibility more consistent with actual travel.

## 6. Conclusions

In this research, we take the estimation of accessibility as the research object and establish a scientific model to calculate the comprehensive accessibility of an area of urban land. An example of Zhicheng is also carried out as a verification analysis.

In the estimation model, we firstly divide the urban land into grids and process the road network to make it correspond to the model of grid. Comprehensive accessibility of each grid consists of this grid’s external accessibility and internal accessibility. Travel time between grids and the importance level of every grid are needed to calculate the external accessibility. When it comes to the internal accessibility, we use an indicator of road density to describe the internal accessibility of one grid. Lastly, the comprehensive accessibility of every grid in the urban land can be estimated in a batch based on the result of external and internal accessibility calculated above. We use Geographic Information System in this research to realize the process of calculation and the visualization of results.

By taking Zhicheng as an example, the proposed model reveals its excellent effect. The results indicate that the gradual spatial changes in the comprehensive accessibility of the grids is highly consistent with the spatial development trend of the urban land, which verifies the feasibility and accuracy of the measurement method of the comprehensive accessibility. This case study shows that the model proposed in this article can be used in urban planning practice to guide the location selection of important public service facilities.

In the future, we may do more investigations, and the travel behavior of residents can be more consistent with actual situation by using related statistical results. Therefore, the estimation accuracy will be improved with various aspects of data.

## Figures and Tables

**Table 1:** Advantages and disadvantages of four types of accessibility estimation methods.

Estimation Method	Metrics	Advantage	Limitation	Representative
Based on spatial barrier	Physical isolation, time or cost	Easy to understand	Consideration given only to space barriers without considering other factors	Baxe and Lenzi [7]
Based on topological structure	Number of connected nodes	Easy to understand and calculate in batches	Consideration given to the number of nodes that can be connected without considering the cost of reaching a connected node	Taaffe and Gauthier [8]
Based on gravity model	Combination of field distance and attraction measurement	Comprehensive consideration, different weights assigned to each location through attraction measurement, which is closer to the real situation	Subjectivity in calculating gravity	Hansen [4]
Based on accumulative opportunities	The number of places that can be reached at a given time or distance	Easy to calculate and understand	The arbitrariness of time (or equidistant) selection; there is no difference between a place adjacent to the origin and a place that happens to be within the same time range	Sherman [9]

**Table 2:** List of ArcGIS data layers.

Point Data	Line Data	Surface Data
Medical service	Expressway	Island
Leisure and entertainment	Highway	Sea area
Township	Arterial Road	Green space
County	Urban road	Provincial area
Parking lot	Rural road	City area
Provincial capital	Branch road	Water system
Bus stop	River (level 1–5)	County area
Car service	Railway	Township area
Other facilities	Pedestrian path	
Tourist attraction	Ferry route	
Research and education	Light rail	
Financial service		
Population distribution data		
Government agencies		
Gas station		
Airport		
Railway station		
Shopping mall		
Police station		
Highway service station		
Building		
Village		
Food		
Lodging		

**Table 3:** Speed limit for various levels of roads.

Road Level	Speed Limit
Expressway	Fill by attribute field
Highway	80 km/h
Arterial road	60 km/h
Urban road	40 km/h
Rural road	40 km/h
Branch road	18 km/h

**Table 4:** Time required for the path between the center points of each grid.

Name	OriginID	DestinationID	DestinationRank	TotalTime (h)
Point 1–Point 1	1	1	1	0
Point 1–Point 2	1	2	15	0.079044893
Point 1–Point 3	1	3	20	0.087697539
Point 1–Point 4	1	4	25	0.094061091
Point 1–Point 5	1	5	123	0.159255227

**Table 5:** Correlation analysis of internal accessibility and external accessibility.

		Internal Accessibility	External Accessibility
Internal accessibility	Pearson correlation	1	0.408 *
	Significance (two-sided)		0.000
	N	1669	1669
External accessibility	Pearson correlation	0.408 *	1
	Significance (two-sided)	0.000	
	N	1669	1669

* Significantly correlated at the 0.01 level (two-sided).
